# Ectomycorrhizal fungal communities of Swiss stone pine (*Pinus cembra*) depend on climate and tree age in natural forests of the Alps

**DOI:** 10.1007/s11104-022-05497-z

**Published:** 2022-05-26

**Authors:** Edoardo Mandolini, Margit Bacher, Ursula Peintner

**Affiliations:** Institute of Microbiology, University of Innsbruck, Technikerstrasse 25b, 6020 Innsbruck, Austria

**Keywords:** Alpine timberline ecotone, Cool and dry forests, Slope exposure, *Suillus*, *Rhizopogon*

## Abstract

**Background and Aims:**

*Pinus cembra* represent a typical and important tree species growing in European subalpine and alpine habitats. The ectomycorrhizal (ECM) fungal communities associated to this tree under natural conditions are largely unknown.

**Methods:**

In this study, we investigated the ECM fungal abundance and composition at four high-altitude sites (two northern-exposed and two southern-exposed habitats) in South Tyrol (Italy), and included also two different age classes of *P. cembra*. The ECM partners were characterized morphologically, and identified by rDNA ITS sequence analysis.

**Results:**

The degree of mycorrhization in adult *P. cembra* was typically 100% in these natural habitats, with a total species diversity of 20 ECM species. The four high-altitude sites were similar concerning their species richness and mycobiont diversity, but they differed significantly in ECM species composition. Young *P. cembra* had a mycorrhization degree of 100% and a total of 10 species were observed. All mycorrhizal partners of naturally regenerated young *P. cembra* were only detected in one specific location, with the exception of *Cenococcum* sp. and *Amphinema* sp. which were detected at two sites. Young trees harbour a distinct ectomycorrhizal fungal diversity, which is clearly lower than the diversity detected in adult *P. cembra* trees. The *P. cembra* bolete (*Suillus plorans*) is the most important symbiotic partner of *P. cembra* at Southern Tyrolean high-altitude sites and is known for its strict, species-specific host association.

**Conclusions:**

The ectomycorrhizal fungal community composition strongly depends on geographic region and on the slope exposure (north or south) of the site.

**Supplementary Information:**

The online version contains supplementary material available at 10.1007/s11104-022-05497-z.

## Introduction

*Pinus cembra* L. (Swiss stone pine) is a tree species typical of subalpine / alpine European habitats that is often considered a glacial relict of high conservation value. This five-needle pine is well adapted to high-altitude ecosystems within an elevation range between 1500 and 2500 m above sea level (a.s.l.) (Casalegno et al. 2010) where harsh environmental conditions like low winter temperatures, elevated ozone levels, and radiation persist. *Pinus cembra* is an important tree species of the Alpine timberline ecotone (Ulber et al. 2004; Apetrei et al. 2011) where growth is clearly limited by temperature. In fact, this tree does not survives in areas with mean daily air temperatures above 6–8 °C during the vegetation period (Oberhuber 2004; Rossi et al. 2007) giving a competitive advantage over *Picea abies*, which otherwise dominates the subalpine vegetation zone of the Alps and the Prealps. The substratum type is not particularly significant for *P. cembra*, which grows both in calcareous or siliceous conditions. The bird *Nucifraga caryocatates* acts as the main dispersal vector of *P. cembra* seeds (Caudullo and de Rigo 2016), and may play an important role in the upward shift of the tree due to increasing rates of global warming (Holtmeier and Broll 2005).

*Pinus cembra* trees are usually strongly mycorrhized (Keller 1989; Göbl and Ladurner 2000). The host specificity of fungal symbiotic partners is highly variable: generalists form symbioses with a wide variety of plants, whereas specialists associate with plants of only one genus or species. The advantage of host-specialism lies in the highly efficient nutrient and water transfer between the symbiosis partners, and in the exclusion of mycoheterotrophy (Simard et al. 2012). Therefore, host-specialists are often the dominating ectomycorrhizal (ECM) species in high-altitude areas with extreme conditions (Moser 1958; Heumader 1992). Three basidiomycete species of the genus *Suillus* form a strictly host-specific symbiosis with *P. cembra* trees in these regions: *S. plorans*, *S. placidus,* and *S. sibiricus* (Göbl and Ladurner 2000; Bacher et al. 2010; Rainer et al. 2015). They belong to the largest monophyletic group of ECM fungi that occur exclusively in symbiosis with a single plant family (Pinaceae) (Zhang et al. 2022). These three *Suillus* species occur in the alpine region exclusively with Swiss stone pines and are therefore called "Swiss pine boletes". The Swiss pine boletus (*S. plorans* (Roll.) Sing.), the ivory boletus (*S. placidus* (Bon.) Sing.) and the ringed Swiss pine boletus (*S. sibiricus* (Sing.) Sing.) differ physiologically by different site and nutrient requirements (Keller 1996). *Suillus* mycorrhizae are characterized by their initially coralloid, then nodule-like form, and extensive mycelial network attached to the mycorrhiza (Göbl and Ladurner 2000; Rainer et al. 2015). With thick strands of hyphae (rhizomorphs), *P. cembra* boletes traverse the substrate and cover long distances in search of nutrients and water, thus they are considered to be of the "long-distance exploration type" according to Agerer (2001). Due to host specificity, these mycelial networks exclusively connect plants of the same species (Kennedy et al. 2007), potentially allowing nutrient translocations between different tree individuals (e.g., from old to young stone pines). Host specialization prevents material flows to other tree species (interspecific epiparasitism / mycoheterotrophy). Besides *Suillus* species host-specialist, there are a number of generalist ECM fungi that are associated with *P. cembra.* For example, *Amphinema byssoides*, *Thelephora terrestris* or *Cortinarius* species*,* are ECM symbionts whose function ranges from trophic interactions to protective effects (Selosse et al. 2004), but they are usually occurring in low abundance only (Kranabetter 2004; Rainer et al. 2015). The mycobiont species composition can change during the tree development, and seedlings may have a different species composition than adult trees (Visser 1995; Trocha et al. 2006; Bacher et al. 2010).

Up to the present, the knowledge concerning ECM communities of *P. cembra* is either based on empirical data concerning fruiting body occurrence (Favre 1960; Horak 1963; Keller 1989), mycorrhizal morphotyping (Göbl and Ladurner 2000), or ECM communities from afforestations (Moser 1963; Rainer et al. 2015) and nurseries (Bacher et al. 2010). The ECM fungi associated to *P. cembra* in an afforestation in North Tyrol (Austria) were long-term monitored, with the aim of confirming the sustainability of ECM inoculation with Swiss pine boletes, which had been carried out in 1960. The results documented only small differences in the species composition between young and adult trees in this afforestation, with the ECM communities strongly resembling the original *Suillus* species inoculum (Göbl and Ladurner 2000; Rainer et al. 2015). However, the ECM communities of young and adult *P. cembra* trees in natural forests remain largely unknown. Thus, this study aims to identify the ECM symbiotic partners of *P. cembra* in different natural Southern Tyrolean forests. Specifically, we wanted to ask the following questions: (i) What is the ECM status of natural Swiss stone pines? (ii) Are there different ECM fungal communities characteristic for the different age-stages? (iii) Are there ECM fungi characteristic for *P. cembra* habitats in general, or are there fungal indicator species adapted to specific habitat conditions? We hypothesize that some ECM partners are ubiquitous host-specialists to *P. cembra,* but that other fungal partners are specific to the location, environment and climatic conditions.

## Materials and methods

### Study site description

**Fig. 1:**
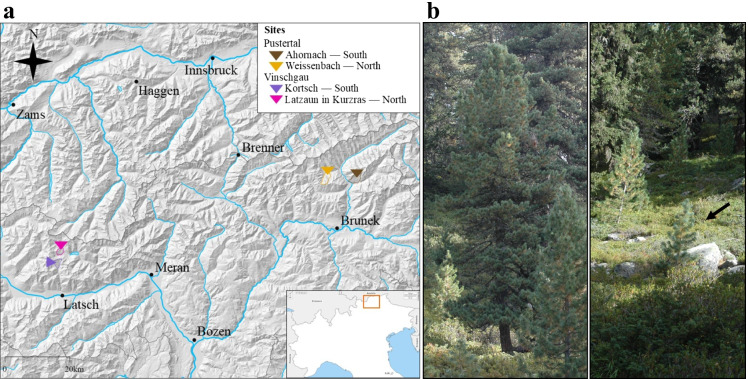
**a** Southern Tyrolean study region including the four sampling sites for the investigation of the ECM communities of *Pinus cembra.* The sampling sites are marked: Pustertal southern-exposed Ahornach—dark brown; Pustertal northern-exposed Weißenbach—light brown; Vinschgau southern-exposed Kortsch -purple; Vinschgau northern-exposed Latzaun—light purple. The slope area of the sites is also marked on the map. **b** *P. cembra* trees of different age stages investigated during this study: adult individuals (left) and young individuals (right—arrow)

Two different regions in South Tyrol (Italy) were chosen to carry out the study on ECM fungal communities in *P. cembra* root systems, the Pustertal and Vinschgau. These regions have an opposite geographical location in South Tyrol with overall different climate due to wind exposure, annual precipitations, and historical development. For each valley, two different high-altitude *P. cembra* forest sites were selected (Fig. 1a, Table 1): one on a southern-exposed slope, and one on a northern-exposed slope. Because of the sun direction, slope exposure is an important variable when considering the local climate of the sites: southern slopes are usually drier and warmer, while northern slopes are cooler and moister.

**Table 1:** Study sites for the investigation of ECM communities associated to natural *P. cembra* forests in South Tyrol, with location, coordinates, altitude, face orientation of the slope (N = north, S = south), annual mean precipitation, mean temperature during the vegetation period, soil pH, and soil type

Sites	Ahornach	Weißenbach	Kortsch	Latzaun
Location	Pustertal		Vinschgau	
Position	46.9355556, 11.9905554	46.9305556, 11.90277778	46.6966667, 10.7672223	46.7575000, 10.7702778
Altitude	2000 m	2000 m	2000 m	2100 m
Exposition	S	N	S	N
Vegetation	Subalpine	Subalpine	Subalpine	Subalpine
Precipitation.^a^	917 mm		660.1 mm	
Temperature.^b^	5.4 ± 8.4		3.4 ± 7.2	
pH	4.1	4.1	4.6	4.4
Soil type	Semipodsole	Semipodsole	Semipodsole	Semipodsole

^a^Yearly, ^b^Annual mean in 2007

In Pustertal, the area in Ahornach represents the southern-exposed site whereas the area in Weißenbach the northern-exposed one. In the former, the average age of *P. cembra* was about 80 years, with individual spruces interspersed in the stand. In the latter, the average tree age was about 50–80 years, with single spruces intermixed. In the region of Vinschgau, Kortsch near Schlanders represents the southern-exposed site. Here, the average age of the *P. cembra* was about 80 years, with interspersed spruces. The northern-exposed forest is located in Latzaun near Kurzras. The average age of this pure *P. cembra* stand was about 50 years. In all locations, sampling was carried out at the same altitude, at roughly 2000–2100 m above sea level.

The sampling area is typically free of snow between April or May and October or November. Detailed information on the soil properties (https://tirolatlas.uibk.ac.at/maps/interface/topo.py/index?image=c7_boden), mean year temperature, and total year precipitation (https://meteo.provincia.bz.it/default.asp) of the sites are reported in Table 1. Soil samples were taken at each site for pH measurements in 0.01 M CaCl2 as described in Thomas (1996) (Table 1).

### Sample collection and mycorrhizal morphotyping

Within each site, two different age stages of *P. cembra* were identified, namely adults spruce (Adult) and young spruce (Young) based on their height and stem diameter (Fig. 1b).

To sample the ECM fungal community on the root system of adult pines (between ten and fifteen m high) in each of the four sites, 5 soil blocks were randomly taken with a spade in the *P. cembra* area. The soil blocks had an area of 20 × 20 cm and depth varying between 10 and 20 cm, depending on the subsoil conditions. Care was taken to ensure that the distance to interspersed spruce was as great as possible. Sampling of all sites took place during the last week of September and the first week of October 2008. In order to determine the autochthonous ECM fungal community in young pines, 10 whole individuals were retrieved from each of the four sites in mid-June 2009. The plants were between five and fifteen cm high, and had a maximum stem diameter of 6 cm.

The root systems retrieved from the young stone pines and from the soil blocks were gently washed in tap water over a 2 mm sieve to remove most of the soil and organic debris by hand, minimizing damage to the ectomycorrhizas. Material, which was adhering tightly to the root system, was removed with forceps. All root systems from each stone pine and soil block were distributed in Petri dishes and then a specific number of mycorrhized fine roots were randomly taken and counted. A total of 600 root tips were analysed for each age class and site. The number of analysed root tips associated with young plant varied between 30 and 300.

All observed root tips were distinguished based on morphological criteria following Agerer (2001) and classified into different morphotypes (MT) using a Nikon SMZ800 stereo-microscope at 10—to 100—fold magnification. The following parameters were decisive: colour of the root tip, surface texture (e.g., smooth, granular, tomentose, hairy), presence of detached hyphae or a hyphal web, structure and length of the mycorrhizal axis. For each MT, at least 4 root tips were selected and stored at -20 °C in Eppendorf-tubes containing 50 μl cetyltrimethyl ammonium bromide (CTAB) buffer until DNA extraction. The remaining root tips were placed in 200 μl of 70% ethanol and stored at 4 °C until further processing. Genomic DNA from MT which did not have at least 4 root tips was not extracted, thus not sequenced, and were categorized as “Rare Mycorrhizal tips”.

### DNA extraction, amplification and sequencing

Genomic DNA was extracted from individual root tips following Southworth (2000; http://www.sou.edu/ BIOLOGY/Faculty/Southworth/CTAB.htm). For DNA amplification, the following primer combinations were used: ITS1F × LR15, ITS1F × LR21, ITS1F × NL4 and ITS1F × ITS4. Purified PCR products (ExoSAP-IT PCR Clean-up Kit, GE-Healtcare Europe GmbH, Austria) were sent to Genoscreen (Lille, France) for sequence analyses for the ITS1 region. Details for DNA extraction and molecular amplification can be found in Bacher et al. (2010).

### Data analysis and statistics

Resulting rDNA ITS sequences were edited and checked using Sequencher (version 4.6; Gene codes Inc. Ann Arbor, MI). Sequences were merged and clustered into operational taxonomical units (OTUs) with a 97% score similarity based on greedy clustering algorithm (agc) using MOTHUR v1.44 (Schloss et al. 2009). Afterwards, the taxonomic affiliation of each unique sequence was obtained using UNITE Fungal ITS train set 10–05-2021 (Abarenkov et al. 2021) with kmers analysis (1000 interactions) on a confidence bootstrap threshold of 80% (classify.seqs). Then, UNITE and GenBank databases (Clark et al. 2016) were used to determine the best match sequence based on the taxonomic affiliation and bootstrap values (Kõljalg et al. 2005) (Supplementary Table S1). Species belonging to the *Suillus* genus frequently present higher variability in the ITS regions, while still belonging to the same morphological concept species based on the fruiting body material (ID: IB20050424) (Zhang et al. 2022). Thus, when distinct OTUs matched to the same best match sequence in the database, they were annotated according to the best match sequence classification, and distinguished using numbers (e.g., *Suillus plorans*_1).

Relative occurrence frequencies were calculated by counting how many times a taxon was found across individuals of the same sample (5 individuals for adults, 10 individuals for young). Frequencies are reported in percentage. In the heatmap, clustering analysis for the categorical factors are based on Euclidean distances.

Abundances of morphotypes were converted to abundances of OTUs based on molecular identification. The structure of the ECM fungal community based on counts of each OTU from each individual (biological replicates) across all samples was analyzed by non-metric multidimensional scaling (NMDS), using the Bray–Curtis distance measure to generate the dissimilarity matrix with the R package “vegan” (Oksanen 2015). The amount of variance explainable by the experimental factors (Location and Age) on the ECM fungal community was calculated using permutational multivariate analysis of variance (adonis) function (999 permutations) on Bray–Curtis distance matrix (Oksanen 2015).

For each age stage within each site, proportionate abundance matrices were generated by averaging the number of MT of each OTU across tree replicates and diving it by the total number of MT in each sample. This resulted in an OTU table listing the relative abundance of all the detected OTUs in all of the samples. Alpha diversity indexes and relative abundance graphics were carried out with relative abundance of OTUs. The ECM fungal diversity was expressed using taxa richness, evenness and Shannon’s diversity index (H′) at species level, calculated from the overall relative abundances of taxa for each site and age stage.

All the statistical analyses and graphs (ggplot2) were performed in R (version 4.0.3) (R Team 2013).

## Results

### ECM fungal diversity increases with tree age and differs among forest locations

**Fig. 2:**
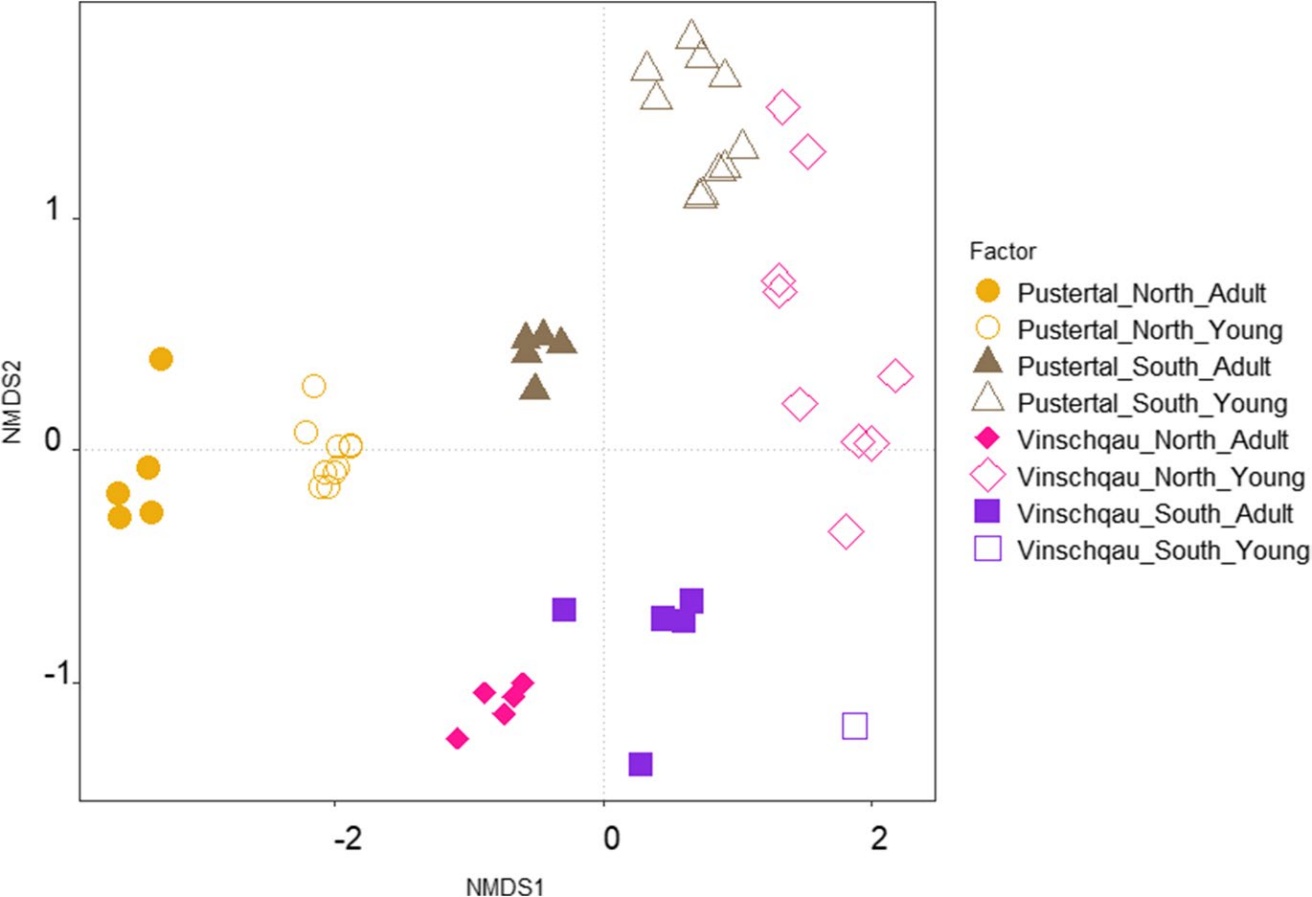
Nonmetric multidimensional scaling (NMDS) analysis based on Adonis and Bray–Curtis distance matrix on the OTUs sequences of the ECM fungal community in different *Pinus cembra* sites. Stress value = 0.0289. Total R^2^ = 0.681. Points closer together in the ordination space have more similar community assemblages

**Fig. 3:**
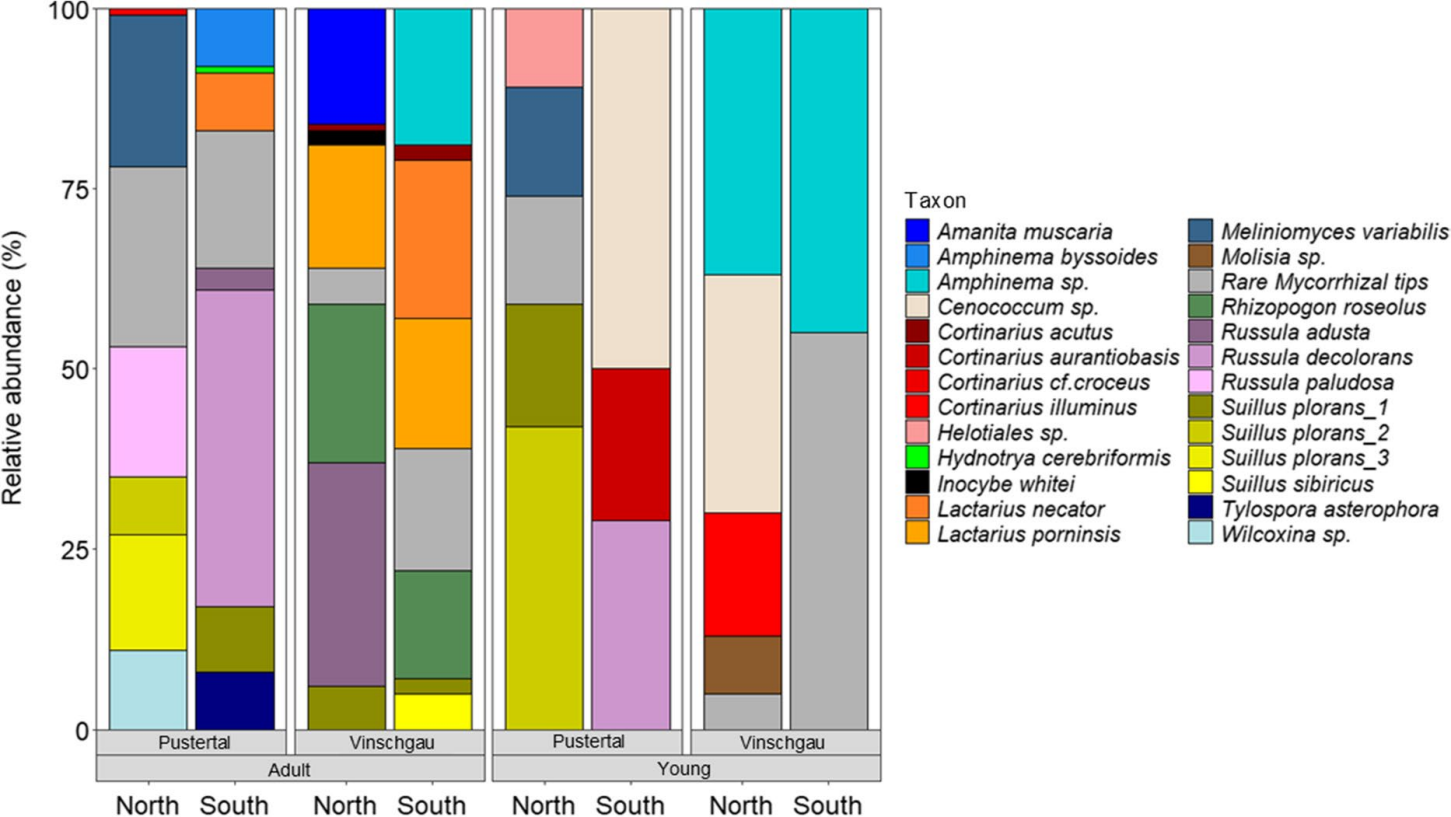
Relative abundance of ECM fungi at the species level compared between adult and young *P. cembra* trees in the different sampling regions (Pustertal, Vinschgau) and sites (representing the slope exposure north, south)

**Fig. 4:**
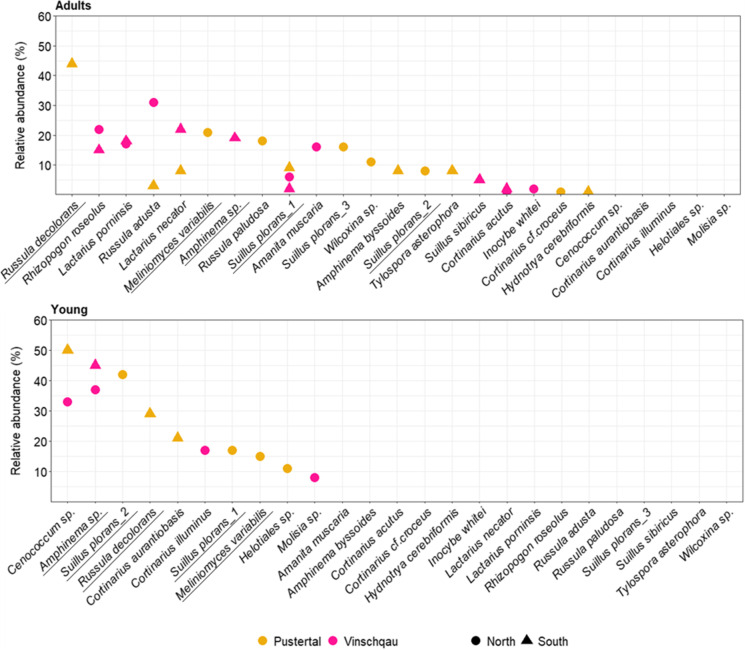
Differential representation of significantly abundant ECM taxa found in the *P. cembra* roots of adult (above) and young (below) *Pinus cembra* trees. On the x axis, ECM species are ordered based on their overall abundances across all four sites. For each species, dots represent their relative abundance (%) coloured by region (Pustertal or Vinschgau), and labelled by site (representing slope exposure north or south). Taxa found in both the adult and young trees are underlined

Both adult and young trees had a mycorrhization level of 100%. A total of 31 different root morphotypes were differentiated from *P. cembra* roots of both adult and young trees across all four sample sites. After OTU clustering and taxonomic assignment, a total of 25 unique ECM fungal taxa were identified (Fig. 3). We found no significant changes in total fungal species richness between Pustertal and Vinschgau regions, while there was a strong difference in richness between adult and young trees (F_(2,1)_ = 32, P = 3e-04, Table 2). Moreover, there was a difference only within young trees, between northern and southern slopes (F_(2,1)_ = 4.5, P = 0.01, Table 2). On average, ECM species richness in young trees was almost half-fold lower than in the adult trees (p = 0.001). Twenty ECM taxa were found belonging to adult trees across all sites, while 10 taxa in the young trees. A total of 15 species were unique to the adult and a total of 5 to the young trees (Fig. 4). In addition, beta diversity analyses showed that the species composition of each high-altitude site was relatively homogeneous within tree age, but sites differed greatly from one another (Fig. 2).

**Table 2:** Diversity indices for the mycorrhizal fungal communities associated with *P. cembra* (richness, evenness, Shannon’s diversity (H)) for all sites. Different letters indicate significant differences between factors at p < 0.05 (Tukey’s test)

Sites	Richness	Eveness	Shannon (H)
Pustertal_North_Adult	7^a^	0.908108	1.767097^a^
Pustertal_South_Adult	8^a^	0.793919	1.650909^a^
Vinschgau_North_Adult	8^a^	0.833639	1.733504^a^
Vinschgau_South_Adult	8^a^	0.889363	1.849379^a^
Pustertal_North_Young	5^b^	0.918034	1.477519^a^
Pustertal_South_Young	3^c^	0.940544	1.033293^b^
Vinschgau_North_Young	5^b^	0.861673	1.38681^a^
Vinschgau_South_Young	2^c^	0.992774	0.688139^b^

ECM fungal composition differed significantly between regions, slope exposure, and age of the trees (Fig. 2). Bray–Curtis distance analyses indicated that regions (Pustertal vs. Vinschgau) imposed the largest effect on community composition, accounting for 17% of the variation in the ECM fungal community composition (R^2^ = 0.17, p = 0.001). Additionally, sites, the combinatory factor of regions and slope exposure (north vs south), accounted for 15% of the total community composition (R^2^ = 0.15, p = 0.001). The tree age (adult vs. young) was also a strong explanatory factor, explaining 12% of the variability (R^2^ = 0.12, p = 0.001), followed by slope exposure with 10% (R^2^ = 0.102, p = 0.001). The interaction between regions and age, as well as the interaction between age and exposure, accounted for 8% (R^2^ = 0.0802, p = 0.001) and 5% (R^2^ = 0.049, p = 0.001) of the variability, respectively. The ECM communities associated with young *P. cembra* trees tended to cluster together and are significantly different from all other ECM communities of adult trees. Interestingly, the ECM community in young and adult trees of the northern site of Pustertal clustered closer to each other than to the ECM assemblages of other sites.

### A few, different taxa dominate the ECM fungal community in each forest site

The most abundant species in adult *P. cembra* trees in the Pustertal valley were *Russula decolorans* (44%), *Meliniomyces variabilis* (21%), *Russula paludosa* (18%), and *Suillus plorans_*3 (16%). Sampling at the southern-exposed site Ahornach revealed a mycorrhizal degree of 100%, with a total of 13 morphotypes distinguished and 7 fungal partners identified (Fig. 3). The average number of morphotypes found in a soil block of was 6.40 ± 1.15 per L soil. The most frequent mycorrhizal partners in this site were *Russula decolorans* (44%), *Suillus plorans_*1 (9%), *Amphinema byssoides* (8%), *Lactarius necator* (8%), and *Tylospora asterophora* (8%). Sampling at the northern site-exposed Weißenbach revealed a mycorrhizal degree of 100%, with a total of 14 morphotypes distinguished and 6 fungal partners identified (Fig. 3). The average number of morphotypes found was 4.60 ± 0.55 per L soil. The most frequent mycorrhizal partner was *Melinomyces variabilis* (21%), followed by *Russula paludosa* (18%), *Suillus plorans_*3 (16%), *Wilcoxina* sp. (11%), and *Suillus plorans_2* (8%).

The most abundant species in adult trees in Vinschgau valley were *Rhizopogon roseolus (37%)*, *L. porninsis* (35%), and *R. adusta* (31%). Sampling at the southern-exposed site Kortsch revealed a mycorrhization level of 100%, with a total of 13 morphotypes and 7 fungal partners identified (Fig. 3). The average number of morphotypes detected in a soil block was 4.20 ± 0.45 per L soil. The most frequently detected taxon was *L. necator* (22%). Other important mycobionts were *Amphinema* sp. (19%), *L. porninsis* (18%), and *R. roseolus* (15%). Sampling at the northern-exposed site Latzaun revealed a mycorrhization level of 100%, with a total of 11 morphotypes distinguished and 7 fungal partners identified (Fig. 3). The average number of morphotypes found was 5.00 ± 0.71 per L soil. The most frequent mycorrhizal partners were *R. adusta* (31%), *R. roseolus* (22%), *L. porninsis* (17%), and *A. muscaria* (16%).

In contrast to the ECM fungal community of adult trees, the mycobionts composition on young trees was much simpler. In Pustertal valley, *Cenococcum* sp. (50%), *S. plorans_*2 (42%), *R. decolorans* (29%), and *Cortinarius aurantiobasis* (21%) were the most abundant mycorrhizal partners of the trees. In the southern-exposed site Ahornach, three species could be distinguished: *Cenococcum* sp. (50%), *R. decolorans* (29%), and *C. aurantiobasis* (21%). On average, 2.20 ± 0.42 of morphotypes per plant were detected. In the northern site Weißenbach, 4 species could be distinguished, with on average 3.60 ± 0.52 morphotypes per plant (Fig. 3). The most frequently detected mycorrhizal partner was *S. plorans_*2 (42%). *Suillus plorans*_1 (17%), *Melinomyces variabilis* (15%), and *Helotiales* sp. (11%) were also very abundant.

In the young trees of Vinschgau valley, *Amphinema* sp. (82%) dominated the stone pine roots in both the northern and southern slopes (37% and 45%, respectively). In the southern-exposed site Kortsch, 6 morphotypes and only 1 species could be distinguished, with an average of 2.80 ± 0.92 morphotypes per plant detected, while in the northern-exposed site Latzaun 6 morphotypes and 4 species could be distinguished, with an average of 1.40 ± 0.51 morphotypes per plant (Fig. 3). In this site, also *Cenococcum* sp. (33%), *Cortinarius illuminus* (17%), and *Molisia* sp. (8%) were also identified.

Little resemblance was found across sites when considering the species compositions of the trees (Fig. 4). The few exceptions regarding the northern-exposed site of Pustertal, where *M. variabilis* and *Suillus plorans_2* were found both in the adult and in the young trees in high abundances. In the southern-exposed site, only *Russula decolorans* was shared between different tree ages. No taxa were commonly found both in the northern-exposed and in the southern-exposed slopes, at any age stages. ECM communities in Vinschgau were the most distinct. In fact, with the exception of *Amphinema* sp., no taxa were found both in the adult and in the young trees, at any slope exposure. Nevertheless, there was a stronger similarity in species composition between the northern and southern sites in Vinschgau of adult trees compared to Pustertal, with at least three species found on both slope types: *Rhizopogon roseolus*, *L. porninsis*, and *S. plorans*_1. Across regions, very few ECM species were detected both in Vinschgau and Pustertal (Fig. 4). In the adult trees, *Russula adusta*, and *S. plorans*_1, while only *Cenococcum* sp. was found in both regions in the younger trees. *S. plorans*_1 was the most frequently and abundantly occurring mycobiont out of 25 fungal species associated to *P. cembra* at both regions.

### Local distribution of ECM partners depends on the forest location site

**Fig. 5:**
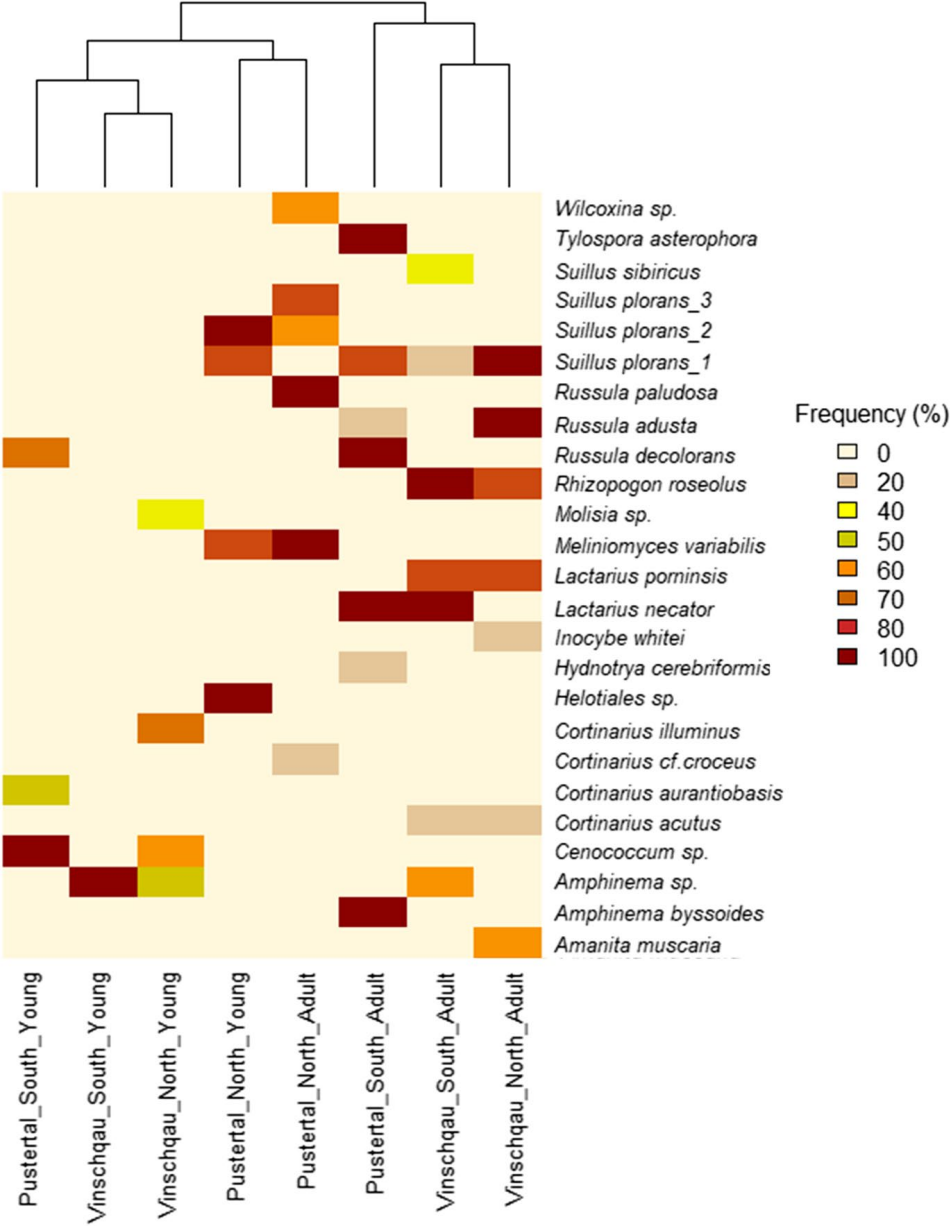
Heatmap and accompanying cluster analysis (x axis) of the relative occurring frequency of ECM fungal taxa across *Pinus cembra* of the same site (regions: Pustertal, Vinschgau; face: north, south) and age stage (adult, young). The occurrence frequency for each taxon across sampled trees is coloured in the shades of light yellow (low frequency) to red (high frequency). The number of times each taxon was detected across replicates is given by the frequency percentage, spanning from 100%, taxa detected in all the replicates, to 0%, taxa never detected

Cluster analysis showed that the ECM fungal communities converged into three main clusters. Sites in Vinschgau and the southern-exposed site of Pustertal clustered together in two separated groups based on the age of the tree, while the ECM communities of Pustertal northern-exposed site grouped together for both adult and young tree communities, thus showing a unique and independent community structure (Fig. 5). In fact, beside *S. plorans*_1, the mycobiont assemblage of the tees was distinct for each location.

The spatial distribution of most ECM partners across *P. cembra* individuals within a single location was highly wide-spread (Fig. 5). In fact, the majority of taxa (e.g., *Suillus* spp., *Lactarius* spp., *Rhizopogon* sp.) were found on more than 60% of the adult individual trees. *Cortinarius* spp., *Hydnotrya cerebriformis*, *Inocybe whitei*, *Russula adusta, Molisia* sp.*,* and *S. sibiricus* were found in less than 50% of individuals, thus showing a very localized distribution across the forest ground.

## Discussion

### Mycorrhizal status of adult Swiss stone pine at natural high altitudinal sites

During the present study, a total of 20 fungal species were identified as fungal partners of adult *P. cembra* at high-altitude natural forests in South Tyrol. Nine fungal species were already reported as ECM partners from a pilot study on ECM fungi occurring on 20–35 years old *P. cembra* afforestation in Haggen (Schmid et al. 2006; Rainer et al. 2015). Thus, a total of 30 mycorrhizal partners of *P. cembra* at such sites are now known.

The ECM symbiosis appears to be important for trees at high-altitude sites. In fact, the *P. cembra* roots investigated show a very high mycorrhization degree, coherent with the afforestation site of Haggen (Rainer et al. 2015). Although species richness, species distribution (evenness), and diversity were very similar among sites (5–9 fungal partners), adult *P. cembra* had unique ECM communities within each site. The *P. cembra* bolete (*Suillus plorans*) was the only fungal partner present in all locations investigated, in the afforestation in Haggen (Rainer et al. 2015), and in the afforestation in Venet near Zams (Moser 1963; Schmid et al. 2006), and thus can be considered the most important and widespread symbiotic partner of *P. cembra* in this area. Several species of the genera *Lactarius*, *Russula,* and of *Rhizopogon* were also frequently found, but usually had a more site-specific distribution.

A comparison with the mycorrhizal partners of a 20–35 years old *P. cembra* forest in the afforestation site in Venet near Zams (North Tyrol, Austria) and in Haggen (North Tyrol, Austria) provides information on the sustainability and suitability of mycorrhizal inoculations. The inoculation of the *P. cembra* in both locations was done with a mixture of the three *P. cembra* bolete species (*S. placidus, S. plorans, S. sibiricus*) (Moser 1958; Göbl 1963). All three species of *Suillus* have persisted in the habitats as *P. cembra* mycorrhizal partners, with *S. plorans* predominating (Schmid et al. 2006; Rainer et al. 2015). The uneven distribution of the different species of *Suillus* sp. is based on their different substrate and nutrient requirements (Keller 1996). Thus, for a successful mycorrhizal inoculation, the correct selection of the inoculated material is crucial. Both the fungal species and the inoculum provenances were carefully and correctly selected for the afforestation in Venet and in Haggen. In fact, they corresponded to the natural mycorrhizal distributions of optimally growing *P. cembra* at high-altitude sites where *S. plorans* and *S. sibiricus* also occurred, with *S. plorans* clearly dominant. *S. placidus* was never detected in our investigation, but its favourable role for the establishment of the tree in these environments cannot be excluded.

The fungal diversity of species detected as ectomycorrhizae on *P. cembra* root tips is within the range of the mycobiont richness as reported for other ectotrophic plants of the same altitudinal zone in the Alps with subalpine /alpine vegetation: for example, 22 species were reported for *Larix decidua* (Leski et al. 2008), 28 for *Picea abies* in Swiss forests (Peter et al. 2001), 39 for *Arctostaphlylos uva-ursi* (Krpata et al. 2007), and 19 for *Salix herbacea* growing in the alpine zone (Mühlmann and Peintner 2008). This diversity of fungi associated to *P. cembra* includes three host specialists (*Suillus* spp.). All other mycorrhizal fungi are either generalists or have a wider host range. Interestingly, our data also indicate that host shifts are comparatively frequent in these subalpine habitats. We detected several fungal species known as host specialists to other plant partners: *Lactarius porninsis* (*Larix decidua*) (Nuytinck and Verbeken 2007), and *Rhizopogon roseolus* (*Pinus mugo, P. sylvestris*) (Kipfer et al. 2012). Host shifts are likely to occur at the distributional margin of one partner. In this case, *L. decidua, P. mugo, and P. sylvestris* habitats are partly overlapping, and the switching to *P. cembra* hosts enables the fungal partner to escape from habitat restriction into a new and larger habitat.

### *Suillus plorans* as a "multi-stage" mycorrhizal partner and important host specialist

The fungi identified as symbionts of *P. cembra* can be divided into three different groups: "early-stage” fungi, "multi-stage” fungi, and "late-stage” fungi (Visser 1995). "Early-stage” fungi form symbioses mainly with seedlings and young plants, while “multi-stage” fungi accompany the host plant from the young stage to old age. In contrast, “late-stage” fungi preferentially occur in the adult stage of the plant (Jackson and Mason 1984). In recent studies, *S. plorans* as well as *S. sibiricus* occurred on *P. cembra* trees of all age classes and can therefore be described as typical "multi-stage” fungi (Bacher et al. 2010; Rainer et al. 2015). However, only *S. plorans* dominated in the Southern Tyrolean *P. cembra* forests investigated.

*Cenococcum* sp., *Cortinarius* sp.*, Amphinema* sp.*,* and *Helotiales* sp. were unique to the mycorrhizal assemblage of the young *P. cembra* investigated, and strongly resembled the ECM communities found on the seedlings of *L. decidua*, *P. abies* and *P. cembra* in a previous study carried out in nursery plants (Bacher et al. 2010). Their presence in middle-aged *P. cembra* afforestation (Schmid et al. 2006; Rainer et al. 2015), makes it difficult to categorize them as “early-stage” fungi. However, they are host generalists and where other trees already exist, their established mycelial network can interact and form symbiosis with the new seedlings in an afforestation. This means that ECM colonization of new seedling roots can occur if other subalpine trees exist on enough in the site.

Furthermore, ECM fungal diversity of *P. cembra* increased with host development, due to the addition of species-rich fungal lineages such as *Lactarius* sp. and *Russula* sp., among others. This is in accordance with the successional pattern of ECM communities in temperate and alpine pine systems, where these species become the major components of the ECM assemblages. *Suillus* sp. and *Rhizopogon* sp. have the predominant role in seedling establishment, while the observed succession of ECM fungi may be driven by the accumulation of organic soil matter as the host develops (Koizumi et al. 2018).

### Are there mycorrhizal partners of *P. cembra* that are characteristic for site conditions?

Soil properties were the major drivers of ECM fungal composition in *Pinus pumila* alpine forests, with soil pH, electric conductivity, and total soil carbon being the most important factors (Koizumi et al. 2018). *Suillus plorans* and *S. sibiricus* are generally optimal partners for naturally-regenerated *P. cembra*, plants and adult *P. cembra*, and they were present at all high-altitude sites investigated. These habitats have a siliceous bedrock and are usually characterized by semipodsole soil type with low pH, suggesting a preferential pH range of these species. *Rhizopogon* spp. and *Suillus* spp. are responsible for the characteristic ECM morphotypes “Knöllchenmycorrhiza” (Göbl and Ladurner 2000), a typical representative of the long-distance mycorrhizal exploration type (Agerer 2001). These mycobionts, together with other highly abundant species occurring in both, natural forests habitats and afforestations (e.g., *Amphinema byssoides*), appear to be especially suitable for this special, very stony habitat with high soil heterogeneity.

In their Japanese multi-location study of alpine *P. pumila* forests, Koizumi and Nara (2020) showed that ECM fungal communities were structured by climate factors (temperature) rather than spatial and soil factors. This is in contrast to other recent studies carried out in the Alps, reporting that soil temperature manipulation in a *P. cembra* (Rainer et al. 2015) and *Picea abies* forest (Schindlbacher et al. 2011) did not cause significant changes neither in microbial biomass nor community composition. We speculate that such changes can probably only be detected during long-term studies.

Due to significant differences in the species composition of ECM partners at all high-altitude sites, we speculate that the fungal distribution is highly dependent on the availability of fungal inoculation present at each site. This is in accordance with other studies showing that ECM fungi are found to be patchily distributed at both large (regional) and small (less than 1 m) spatial scales in natural environments (Downie et al. 2021). In fact, no fungal species characteristic of dry or cool sites could be detected. Nevertheless, our results indicate that *S. plorans* might be negatively affected by drought, or higher mean temperatures, as the occurrence of this important mycobiont is clearly reduced in Vinschgau. In fact, this area is characterized by lower annual precipitations and, together with the southern-exposed sites of Pustertal, may result in generally less humid conditions (lower water availability). Furthermore, an *in-vitro* study on the growth response of drought-stressed *P. sylvestris* seedlings, inoculated with the associated host specialists *S. granulatus* and *Rhizopogon roseolus,* indicated a positive effect of *S. granulatus* on shoot growth that was more pronounced under moist than under dry conditions (Kipfer et al. 2012). Environmental factors are also known to limit the distribution of *P. cembra*, which is then replaced by other tree species. *Pinus cembra* is sensitive to drought stress mainly in lower altitudes. It grows better in cool/humid, deep and well-drained soils (Caudullo and de Rigo 2016). We speculate that shifts in ECM communities might be indirectly speeding up replacement of *P. cembra* specialists in these high-altitude habitats: With warmer and drier climate, the highly efficient *P. cembra* host specialist are replaced by ECM host generalist (e.g., *Cenoccoccum, Wilcoxina*), which facilitate introgression of other plant hosts in the habitat. Furthermore, the increased occurrence of hypogeous *Rhizopogon* species at the costs of epigeous *S. plorans* indicates an adaptation to drier environmental conditions. In fact, the *Rhizopogon* genus is a sister group to the genus *Suillus*, and both are known to be host specific to *Pinaceae* (Grubisha et al. 2002). However, *Rhizopogon* was shown to predominate over *Suillus* sp. in dry coniferous forest habitat types dominated by *P. sylvestris* (Hilszczańska et al. 2019). Finally, our data indicate that *Russula paludosa* might be considered as an indicator species typical for cool sites. In Austria, this species is restricted to sites with an annual mean temperature below 8–9 °C and Podsol or Braunerde soil type (http://austria.mykodata.net/Taxa_map.aspx?qvtaxIdTaxon=317058&). Along with other species belonging to this genus, it is usually distributed in hygrophilous conifer forests (Sarnari 2005).

## Conclusions

*Pinus cembra* trees have a rich and diverse partnership with ECM fungi. This partnership is characterized by the occurrence of typical host specialists like *Suillus placidus* and *S. sibiricus* with species-specific adaptation to *P. cembra*, and *Rhizopogon roseolus*, which is known to be host-specific to several *Pinus* spp. The most distinct ECM fungal composition resulted between young and adult trees, with the latter having higher overall richness and diversity than the young trees.

There are clearly distinct ECM communities clustering individually by site, first, and forming sub-clusters by region and slope-exposure (north and south). The age effect is always present regardless of the environment. This means that, especially for adult *P. cembra* trees, fungal communities are typically similar within the same region, but differ with the slope-exposure. A convergence between dry sites can be indicated by cluster analysis and occurrence of taxa. ECM community establishment probably took decennia in each of these sites (highly heterogenic soil which developed on block terrain). The fungal distribution is also highly depending on the availability of fungal inoculum. The specific ecological roles of the detected fungal taxa remain an open question, which warrants to be addressed by further research on these important but vulnerable high-altitude habitats.

## Supplementary Information

Below is the link to the electronic supplementary material.

Supplementary file 1 (DOCX 22.1 kb)

## Data Availability

The raw sequencing data were deposited in GenBank SRA database (https://www.ncbi.nlm.nih.gov/genbank/) under the accession numbers referenced in the Supplementary Material Table S1.
