# Supplementary material for: Ectomycorrhizal fungal communities of Swiss stone pine (Pinus cembra) depend on climate and tree age in natural forests of the Alps
Source: Plant Soil. 2022 May 26;502(1-2):167–80. doi: 10.1007/s11104-022-05497-z (PMC11420379; doi:10.1007/s11104-022-05497-z)
Supplement: Supplementary file 1 — (DOCX 22.1 kb) [file 11104_2022_5497_MOESM1_ESM.docx]

Table S1 Fungal ITS sequence identification and best match sequence retrieved by the UNITE and GenBank database. The taxonomic affiliation and bootstrap values of each unique sequence using kmers analysis with 1000 interactions are also reported. Sequences were deposited to the GenBank SRA database and their accession numbers are included.

| **Taxon** | **Accession No** | **Taxonomic affiliation (Bootstrap %)** | **Best match species** | **Best match accession No** | **Identity (%)** |
| --- | --- | --- | --- | --- | --- |
| *Amanita muscaria* | HM044570 | *Amanita muscaria (100)* | *Amanita muscaria* | UDB036319 | 96 |
| *Amphinema byssoides* | HM044575 | *Amphinema byssoides (95)* | *Amphinema byssoides* | UDB008257 | 91 |
| *Amphinema sp.* | HM044574 | *Amphinema sp. (100)* | *Amphinema sp.* | UDB027332 | 99 |
| *Cenococcum sp.** | HM044579 | *Cenococcum sp. (100)* | *Cenococcum sp.* | KC986263 | 99 |
| *Cortinarius acutus* | HM044580 | *Cortinarius acutus (100)* | *Cortinarius acutus* | UDB001002 | 99 |
| *Cortinarius aurantiobasis** | HM044581 | *Cortinarius aurantiobasis (100)* | *Cortinarius aurantiobasis* | HM068560 | 99 |
| *Cortinarius cf. croceus* | HM044578 | *Cortinarius sp. (100)* | *Cortinarius croceus* | UDB024920 | 99 |
| *Cortinarius illuminus* | HM044582 | *Cortinarius illumines (100)* | *Cortinarius illuminus* | UDB000683 | 99 |
| *Helotiales sp.* | HM044587 | *Ascomycota (100)* | *Helotiales sp.* | UDB027992 | 90 |
| *Hydnotrya cerebriformis** | HM044591 | *Hydnotrya cerebriformis (100)* | *Hydnotrya cerebriformis* | MH430533 | 99 |
| *Inocybe whitei* | HM044584 | *Inocybe sp. (94)* | *Inocybe whitei* | UDB024923 | 99 |
| *Lactarius necator* | HM044585 | *Lactarius necator (98)* | *Lactarius necator* | UDB003215 | 99 |
| *Lactarius porninsis* | HM044586 | *Lactarius sp. (100)* | *Lactarius porninsis* | UDB0746175 | 100 |
| *Meliniomyces variabilis* | HM044589 | *Meliniomyces variabilis (100)* | *Hyaloscypha variabilis* | UDB028187 | 96 |
| *Molisia sp.* | HM044592 | *Vibrisseaceae (99)* | *Mollisia sp.* | UDB0780679 | 96 |
| *Rhizopogon roseolus* | HM044595 | *Rhizopogon sp. (100)* | *Rhizopogon roseolus* | UDB001619 | 96 |
| *Russula decolorans* | HM044598 | *Russula decolorans (100)* | *Russula decolorans* | UDB037036 | 98 |
| *Russula adusta* | HM044597 | *Russula densifolia (89)* | *Russula adusta* | UDB023484 | 99 |
| *Russula paludosa* | HM044603 | *Russula sp. (100)* | *Russula paludosa* | UDB031535 | 98 |
| *Suillus plorans_1* | HM044605 | *Suillus sp. (99)* | *Suillus plorans* | UDB033942 | Locked |
| *Suillus plorans_2* | HM044606 | *Suillus sp. (100)* | *Suillus plorans* | UDB033942 | Locked |
| *Suillus plorans_3** | HM044607 | *Suillus sp. (100)* | *Suillus plorans* | MN706538 | 99 |
| *Suillus sibiricus** | HM044608 | *Suillus sibiricus (99)* | *Suilllus sibiricus* | MT302575 | 99 |
| *Tylospora asterphora** | HM044610 | *Tylospora sp. (97)* | *Tylospora asterphora* | KP172309 | 85 |
| *Wilcoxina sp.** | HM044616 | *Wilcoxina sp. (100)* | *Wilcoxina sp.* | JN544512 | 93 |

*Best sequence match obtained from the GenBank database
